# Analysis of the outcome of mechanical ventilation in patients with acute renal failure

**DOI:** 10.1186/cc10177

**Published:** 2011-06-22

**Authors:** JAA Neto, RB Fernandes, FB Lima, DA Castro, EB Moura, MO Maia

**Affiliations:** 1 Hospital Santa Luzia, Brasilia - DF, Brazil

## Background

Mechanical ventilation (MV) is a factor that may induce or worsen lung injury and also contribute to the failure of other organs. An early manifestation of multiple organ failure in the ICU is acute renal failure (ARF), with a prevalence ranging from 4 to 16%, which is associated with increased rates of mortality.

## Objective

The aim of this study was to analyze the outcome of mechanical ventilation in patients with ARF in the ICU.

## Methods

This is a retrospective and analytical study that included patients aged >18 years, hospitalized in the ICU of HSL under MV for more than 24 hours, from June 2009 to June 2010. Patients with chronic renal failure were excluded. The AKIN criteria were used to stratify patients into three groups: non-ARF, ARF and dialysis ARF. The variables analyzed were age, gender, APACHE II, length in ICU, length of MV, MV outcome and mortality. Statistical analysis used chi-square and ANOVA, with a significance level of 5%.

## Results

**Figure 1:**
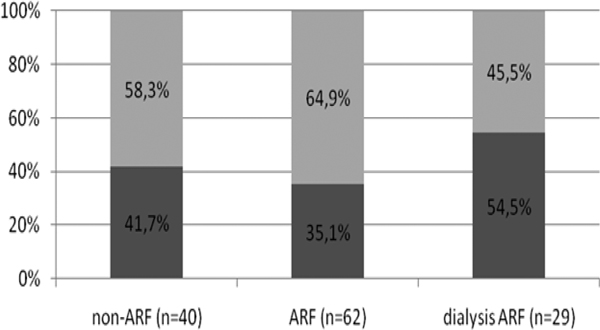
**Distribution of patients who progressed or not to wean from MV**. *P < 0.01.

**Figure 2:**
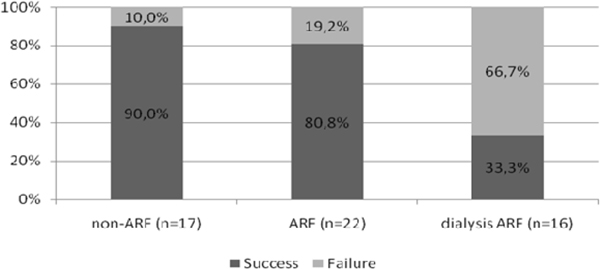
**Outcomes of weaning**. *P < 0.01.

The sample consisted of 131 patients, 51.1% women, mean age 65.6 ± 20.0 years. According to the criterion AKIN, 69.5% of patients had ARF, dialysis was 31.9%. APACHE II was higher in ARF (17.6 ± 7.7) and IRA dialysis (18.6 ± 11.0), compared with the group non-ARF (13.2 ± 7.7), *P *= 0.01. The ICU stay was similar between groups (non-ARF 21.8 ± 32.5 days; ARF 20.8 ± 19.9 days; dialysis ARF 27.1 ± 23.4 days, *P *= 0.53). The duration of MV was higher in the dialysis ARF (non-ARF 5.5 ± 4.7 days; ARF 6.9 ± 7.6 days; dialysis ARF 14.2 ± 15.1, *P *< 0.01). See Table 1 and Figures 1 and 2.

**Table 1:** Characteristics of subjects with AKIN criteria

	No ARF (*n *= 40)	ARF (*n *= 62)	Dialysis ARF (*n *= 29)	*P *value
Age (years)	57.7 ± 20.1	70.3 ± 18.9	66.3 ± 19.1	<0.01
APACHE II	13.2 ± 7.7	17.6 ± 7.7	18.6 ± 11.0	0.01
SAPS II	40.0 ± 14.6	45.6 ± 12.6	44.3 ± 16.4	0.14
Length in ICU (days)	21.8 ± 32.5	20.8 ± 19.9	27.1 ± 23.4	0.53
Length of stay (days)	28.3 ± 36.2	25.2 ± 22.8	29.6 ± 24.0	0.74
Duration of MV (hours)	131.8 ± 112.1	166.7 ± 182.0	341.6 ± 363.4	<0.001

## Conclusions

In the sample studied, we observed a high prevalence of MV and ARF, and the presence of renal failure is associated with a lower success rate of weaning and higher mortality.

## References

[B1] Slutsky AS Tremblay LN Multiple system organ failure. Is mechanical ventilation a contributing factor? Am J Respir Crit Care Med 1998 157 1721 1725 9620897 10.1164/ajrccm.157.6.9709092

[B2] Ranieri M Giunta F Suter PM Mechanical ventilation as a mediator of multisystem organ failure in acute respiratory distress syndrome JAMA 2000 284 43 44 10.1001/jama.284.1.43 10872010

